# Prediction Interval: What to Expect When You’re Expecting … A Replication

**DOI:** 10.1371/journal.pone.0162874

**Published:** 2016-09-19

**Authors:** Jeffrey R. Spence, David J. Stanley

**Affiliations:** Department of Psychology, University of Guelph, Guelph, Ontario, Canada; Universite de Montreal, CANADA

## Abstract

A challenge when interpreting replications is determining whether the results of a replication “successfully” replicate the original study. Looking for consistency between two studies is challenging because individual studies are susceptible to many sources of error that can cause study results to deviate from each other and the population effect in unpredictable directions and magnitudes. In the current paper, we derive methods to compute a *prediction interval*, a range of results that can be expected in a replication due to chance (i.e., sampling error), for means and commonly used indexes of effect size: correlations and *d*-values. The prediction interval is calculable based on objective study characteristics (i.e., effect size of the original study and sample sizes of the original study and planned replication) even when sample sizes across studies are unequal. The prediction interval provides an *a priori* method for assessing if the difference between an original and replication result is consistent with what can be expected due to sample error alone. We provide open-source software tools that allow researchers, reviewers, replicators, and editors to easily calculate prediction intervals.

## Introduction

*“The people who told us about sun block were the same people who told us*, *when I was a kid*, *that eggs were good*. *So I ate a lot of eggs*. *Ten years later they said they were bad*. *I went*, *“Well*, *I just ate the eggs*!*” So I stopped eating eggs*, *and ten years later they said they were good again*! *Well*, *then I ate twice as many*, *and then they said they were bad… Then they said they’re good*, *they’re bad*, *they’re good*, *the whites are good… –make up your mind*! *It’s breakfast I’ve gotta eat*!*”*

Black [1]

The exasperation conveyed by comedian Lewis Black, above, may be similar to what many people experience when reading or hearing about science in the news. Studies with inconsistent findings on the same issue are frequently reported in the media [2,3]. The pain of having to digest inconsistent findings is not isolated to the general public, but can also afflict researchers and their respective fields of study. A number of disciplines–including psychology, biology, neuroscience, and medical research–have reported, what many consider to be, disquieting levels of variability in results [4–10].

Variability in findings across studies can be uniquely troubling when replications are conducted. Because replications are often used to assess the trustworthiness of an effect (e.g., Open Science Collaboration [11]), if an effect cannot be replicated, the finding, and sometimes the associated researchers, may be viewed as questionable. As a result, a replication being interpreted as a success or a failure can have severe consequences for the area of study and perceptions of the researchers’ integrity [12–13]. However, a problem with interpreting replications is that single studies, even if competently executed and properly analyzed, are inherently imperfect pieces of information [14–16]. Specifically, individual studies are susceptible to many sources of error that can greatly influence results [17–18]. By implication, no single study is a pure reflection of the underlying truth. In order to obtain even a judicious estimate of the truth, we must aggregate across a large number of studies on the same phenomena via meta-analysis. As Hunter et al [14] wrote, “Scientists have known for centuries that a single study will not resolve a major issue. Indeed, a small sample study will not even resolve a minor issue. Thus, the foundation of science is the cumulation of knowledge from the results of many studies” (p.10).

If every study contains error, two studies on the same phenomena are likely to differ from the true/population effect as well as from each other in unpredictable directions and magnitudes. Therefore, when interpreting replications, the question is not if deviation across studies is permissible, but instead “how much deviation is permissible?” On this issues, Estes [19] stated, “The principal difficulty with widespread dependence on replication experiments is not that conducting replications is tedious but that judging the success of replications poses an almost intractable problem [20]” (p. 331).

It is becoming increasingly common for researchers to attempt to replicate published effects [11]. There are several large-scale replication initiatives (e.g., “Many Labs” replication project, Reproducibility Initiative, Reproducibility Project: Psychology, Reproducibility Project: Cancer Biology) and many top-level psychology journals are accepting replication articles (e.g., *Journal of Abnormal Psychology*, *Journal of Counseling and Clinical Psychology*, *Journal of Personality and Social Psychology*, *Perspectives on Psychological Science*). As a result, there is a growing need for an objective method for evaluating replication attempts.

One approach for evaluating replications has been to compare the *p*-value of the original study to that of the replication study. An application of this approach involves checking whether the direction of an effect and its classification as statistically significant or statistically non-significant are consistent across the replication and original study. If there is consistency, the replication is deemed successful; however, if there is inconsistency the replication is a failure. Aside from the well-expressed issues of relying solely on *p*-values to interpret results [16, 21–24], Cumming [25] illustrates that this is a flawed approach because *p*-values fluctuate so considerably across replication attempts, due to sampling error, making it a poor criterion to evaluate replications.

Instead of using *p*-values, in rare cases, meta-analytic estimates based on just the original and replicating study have been calculated (see Open Science Collaboration [11] for an example). These meta-analytic estimates are then tested to see if they are different from zero. However, this approach been largely ignored because it is assumed that the effect size estimate from the first study is inflated due to publication bias.

On occasion, confidence intervals have been used to evaluate replications. If interpreted correctly, confidence intervals can offer inferential information not available with *p*-values. That is, confidence intervals provide a plausible range of population values (e.g., population correlations) that could have produced the study result (e.g., study correlation; [26]). Unfortunately, confidence intervals can often be misinterpreted [27–28]. Many misinterpretations involve the incorrect assignment of probabilities to the population parameter rather than the method of constructing the interval (see [28] for a review). However, confidence intervals can also be incorrectly interpreted as representing a capture percentage of study results. This is what Cumming et al [27] referred to as the confidence-level misconception. For example, some researchers may incorrectly interpret a 95% confidence interval for a correlation as indicating the range correlations that can be expected in a replication 95% of the time [27, 29]. Interpreting confidence intervals as being representative of replication probabilities is incorrect and can, therefore, lead to improper interpretations of a replication results.

A useful but lesser-known approach to interpreting replications is to use prediction intervals [25, 30]. In the replication context, this type of interval can be quite informative as it presents a way of quantifying the extent to which a replication study may deviate from an original study. To date, such intervals have been used to estimate what sample means are possible in replications when sample sizes are the same [19, 25]; and have recently been used to reanalyze the results from the Reproducibility Project in Psychology [31]. Prediction intervals can be distinguished from confidence intervals. With confidence intervals, the emphasis is on the interval capturing the population parameter whereas with prediction intervals the emphasis is on capturing future sample statistics [31]. Consider for example, a single population mean and 1000 samples from that population. Constructing a 95% confidence interval for each of the 1000 samples would result in 950 (i.e., 95%) of those intervals capturing the population mean. In contrast, consider a scenario where you have a single original sample mean and then obtain 1000 replication samples. A prediction interval constructed around the original study’s mean would capture 95% of the replication sample means. Thus, confidence intervals are designed to capture population parameters whereas prediction intervals are designed to capture sample statistics [32–33].

Having an interval that indicates the extent to which a future sample statistic (e.g., correlation) may differ from the current sample statistics due to sampling error is incredibly useful in the replication context. For instance, if a replication result falls within the prediction interval it would suggest that the deviation that was observed between the two studies, was not greater than could be expected due to sampling error alone. Conversely, a replication result that falls outside the interval may be indicative of what is often referred to as a “replication failure,” such that the deviation between results was greater than could be expected due to random sampling alone.

In the current paper, we derive methods to calculate prediction interval for means, and commonly used indexes of effect size: correlations and *d*-values, when sample sizes between studies are not equal. Being able to accommodate different sample sizes in the calculations is important for the application of the prediction interval because it is common for replication studies to have different sample sizes than the original study. Previous applications of prediction intervals to replications have largely focused on scenarios where the original and replication sample size were the same [19, 25, 29–30]. Additionally, our methods accommodate skewed sampling distributions [34], which allows us to derive formulas that calculate prediction intervals for standardized mean differences (*d*-values) and correlations (*r*). A key aspect of the prediction interval is that it can be calculated before a replication study is conducted creating an *a priori* statement of expectations [35]. An *a priori* statement of expected results enables planned replications and replication proposals to specify, in advance, the criteria that will be used to evaluate the replication, independent of the results of the replication.

To illustrate the efficacy of the prediction interval, we present tests of the prediction interval’s ability to capture replication results across a number of replication scenarios. To make the prediction interval’s calculation easier and to facilitate its use, we developed an online prediction interval calculator and open source downloadable software that will allow anyone to freely and easily calculate prediction intervals.

## A Conceptual Introduction to Interpreting the Prediction Interval

Because sampling error is central to the calculation of the prediction interval, we begin with a simple demonstration of the effect of sampling error on data that illustrates the conceptual reasoning underlying the interpretation of a prediction interval. The potential sources of error in research are vast and some sources of error are more controllable than others [18, 36–37]. Sampling error, however, is present in every study and produces unpredictable effects on the results of individual studies [18]. Sampling error is created through the random selection of some subjects to participate in research over others. The inability of researchers to recruit sample sizes approaching infinity ensures that sampling error is present in every study [18]. Because of its prevalence and objective nature, we focus exclusively on the influence of sampling error when calculating the prediction interval. Although studies are subject to other sources of error (e.g., imperfect validity, dichotomization of continuous variables, methodological confounds, etc.; [18]) to different extents, these sources of error can be difficult to quantify based on objective study characteristics. Sampling error, however, is readily quantifiable based on a study’s sample size and effect size [17–18, 38–39].

Even when both the original and replication study are obtained from the same sampling distribution, the result of the two studies are expected to deviate from each other and from the population value, due to sampling error. To illustrate the effect of sampling error and its implications for the prediction interval, consider Jane, a hypothetical researcher, who is interested in determining if coffee consumption is related to anxiety levels. To test her question, Jane conducts an observational study where she recruits 100 participants, measures coffee consumption and anxiety levels, and then correlates the two variables. What correlation is Jane expected to find?

**Fig 1:**
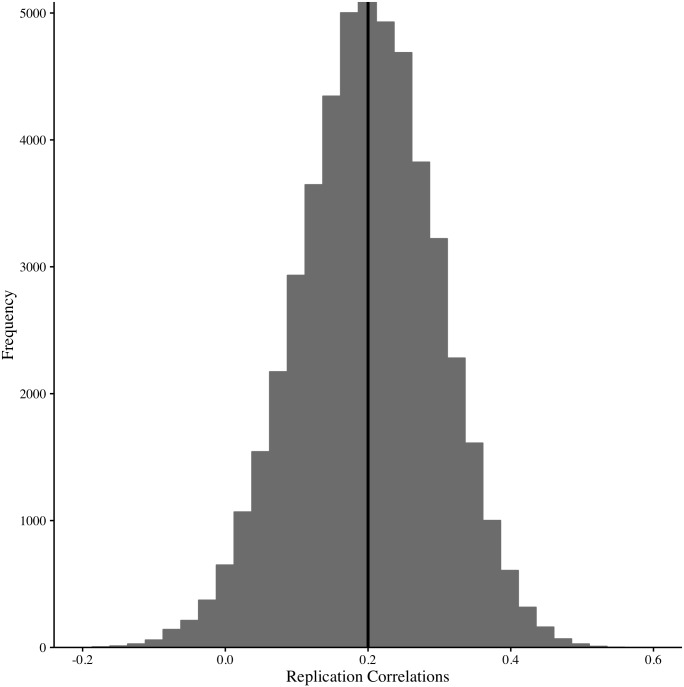
Histograms showing the range of possible study correlations obtained from replications when the population-level correlation is .20. The vertical line indicates the population correlation of .20. The histogram illustrates the results of 50,000 replication studies each with different participants. The variability in study correlations illustrated here is due only to the effects of sampling error.

To understand what correlation Jane is expected to find, it is important to recognize that a population correlation exists between coffee and anxiety and that this population correlation will be reflected in her study correlation. However, because of sampling error, Jane’s result will not be a pure reflection of this underlying population correlation, but merely an estimate of it. To illustrate the extent of the contamination Jane can expect due to sampling error, imagine a scenario where the population correlation is known to be ρ = .20. We can use a computer simulation to obtain 50,000 randomly sampled study data sets (*N* = 100) and obtain a correlation for each of them. Many of these 50,000 study correlations differ from the population correlation (ρ = .20) and from each other due to sampling error. Fig 1 presents the results of the simulation and can be thought of as an empirically derived sampling distribution when the true correlation is .20 and the sample size is 100. The results of the simulation illustrate a wide range of study correlations.

Now let’s imagine that Jane obtained a *r* = .28 correlations in her study as a result that is consistent with what she could expect due to sampling error. She decides to conduct a replication of this study, with the same sample size (*N* = 100), and obtains a correlation of *r* = .07. Even though Jane’s replication correlation (*r* = .07) appears to be substantially different from her first correlation (*r* = .28), inspecting Fig 1 illustrates that the two results are consistent with what can be expected due to sampling error when the population correlation is .20. If, however, Jane’s replication correlation fell outside the histogram, then we know that something other than sampling error may have been responsible for the deviation from the population correlation.

Admittedly, this example is an oversimplification because here we have knowledge of the population correlation, something that is never known. Determining if the two sample correlations differ due to sampling error (i.e., are sampled from the same population) is relatively trivial when the population correlation is known. However, as Stanley and Spence [4] illustrated researchers are continuously faced with the fact that the population correlation is unknown when trying to interpret single correlations. To eliminate some of this inherent uncertainty, we can approach the question from a slightly different perspective. Namely, we can focus on the *difference* between correlations. If pairs of correlations are repeatedly, randomly sampled from the same population, any two randomly selected correlations are expected to differ from each other. However, the average of all the differences will be zero–regardless of whether the population correlation is known or unknown. Conceptually, we could create a histogram of all of the differences (i.e., create a sampling distribution histogram like we did in Fig 1 above). We could then use this histogram to determine what the largest difference could be, in either direction, as a result of sampling error. If a difference between an original and replication correlations is beyond the histogram, then something other than sampling error is likely responsible for the magnitude *difference*. This is the basic reasoning that underlies the interpretation of the prediction interval. In the sections below, we present methods for computing a prediction interval for means, correlations, and *d*-values.

## Prediction Interval for Means

To illustrate how a prediction interval can be computed for means, we will once again consider hypothetical researcher, Jane. Imagine that Jane conducts an original study (*N*_1_ = 50) and obtains a mean *M*_1_ = 98.50 and standard deviation *SD*_1_ = 14.76. She then sets out to conduct a replication study with a sample size twice as large as her original study, *N*_2_ = 100, and wants to know what range of means she can reasonably expect. To answer her question she needs to compute a prediction interval. The prediction interval will tell her how much her replication mean is expected to deviate from her original mean, due to sampling error.

To determine the extent of the difference that we can expect between two random means from the same population (i.e., an original study, *M*_1_, and a replication study, *M*_2_), both of which are affected by sampling error, we need to construct an interval on the difference between two means. That is, we want an interval that describes (*M*_1_—*M*_2_) to determine how large a difference in either direction is possible, as a result of sampling error. To obtain such an interval, we need to know the variance of the (*M*_1_—*M*_2_) sampling distribution. Estes [19] illustrated when the original and replication study have equal sample sizes the variance of (*M*_1_—*M*_2_) can be estimated by $2(SD_{1}^{2}/N)$ (also see [29]). We extend this approach by creating a more generalizable solution, which allows for the possibility of having different sample sizes in the original and replication study. Specifically, we estimate the variance of the sampling distribution of (*M*_1_—*M*_2_) where the sample sizes differ as:

$$\frac{SD_{1}^{2}}{N_{1}}+\frac{SD_{1}^{2}}{N_{2}}$$ (1)

*N*_*1*_ is the sample size of the original study, *N*_2_ is the sample size of the replication study, and *SD*_1_^2^ is the variance of the original study. Because the replication study has not been conducted, we use the variance estimate from the original study (*SD*_1_^2^) as the variance estimate for the replication study. Once the variance of the sampling distribution is obtained using the above formula, the interval for the expected difference between means, due to sampling error, given sample sizes *N*_1_ and *N*_2,_ can be calculated using Eq 2, below. In Eq 2, we take the square root of the variance of the sampling distribution above, resulting in the standard deviation of the sampling distribution (i.e., standard error). In Eq 2, *M*_1_ is the mean observed in the original study, *t* is the two-tailed critical value for *t* when degrees of freedom *df* = *N*_1_-1. Note, that the *df* is not *N*_1_ + *N*_2_−2; this is because only *SD*_1_ is used in the formula instead of a pooled variance. We note that computationally, this prediction interval is the equivalent of calculating a confidence interval for the difference between two sample means (one real, one imaginary and identical to the first), based on the original and replication sample sizes.

$$\begin{array}{c} \text{Mean Prediction Interval}\\ =M_{1}\pm t_{df}\sqrt{\frac{SD_{1}^{2}}{N_{1}}+\frac{SD_{1}^{2}}{N_{2}}} \end{array}$$ (2)

An application of the above formula to Jane’s situation—where her original study’s mean is *M*_1_ = 98.59, *SD*_1_ = 14.76, *N*_1_ = 50, and her replication sample size will be 100 (*N*_2_ = 100)—provides the following solution:

$$\begin{array}{l} 95\%\text{ Mean Prediction Interval}\\ =M_{1}\pm t_{49}\sqrt{\frac{SD_{1}^{2}}{N_{1}}+\frac{SD_{1}^{2}}{N_{2}}}\\ =98.59\pm (2.01)\sqrt{\frac{{\left(14.76\right)}^{2}}{50}+\frac{{\left(14.76\right)}^{2}}{100}}\\ =98.59\pm 5.14 \end{array}$$

This solution, 98.59 +/- 5.14 is an estimate of the range of mean values Jane can expect in a replication that uses a sample size of 100, given her initial study had a sample size of 50. This interval indicates that, due to sampling error, Jane can expect to see a mean as low as 93.45 or as high as 103.73 in her replication study. It is this interval that we call a prediction interval and designate with the following notation 95% PI[93.45, 103.73]. It worth noting that the above solution is a 95% prediction interval because of the *t*-value that was used in Eq 2. A wider (higher percentage interval) or narrower (lower percentage interval) prediction interval can be computed by using larger or smaller *t*-values, respectively.

### Capture Performance Test of the Prediction Interval for Means

In the section below, we empirically demonstrate the ability of the prediction interval to capture 95% of replication means. We also compare the prediction interval’s performance against the 95% confidence interval constructed around the original mean. The script to reproduce our mean capture tests is available at https://github.com/dstanley4/prediction_interval_scripts/.

We tested the prediction interval’s performance using simulated data with the following steps. (1) We obtained a random sample (*N*_1_ = 50), from a population with a mean of 100 and standard deviation of 15 and calculated the “original study” mean (*M*_1_) and standard deviation (*SD*_1_). (2) We computed a 95% prediction interval and a 95% confidence interval around the original study’s mean (*M*_1_), using the methods and formulas presented above. (3) We then simulated the replication study by drawing a second random sample (*N*_2_ = 100) and obtaining the replication mean, *M*_2_. (4) We compared the replication mean (*M*_2_) against the 95% prediction interval and 95% confidence interval to determine if either (or both) captured it. (5) We repeated steps one through five 50,000 times and counted how many prediction intervals and confidence intervals contained the replication mean. Doing so revealed that the 95% prediction interval captured 95.2% of replication means whereas the 95% confidence interval captured 89.7% of replication means.

We then tested different sample size configurations to examine how both the 95% prediction interval and 95% confidence interval performed under different sample size combinations. The results of these tests are reported in Table 1. When sample sizes were equal, the capture percentage of the original study’s 95% confidence interval was roughly 83–84%. It is worth noting that these values are virtually identical to the value of 83.4% reported by Cumming et al [29] who also tested the 95% confidence interval’s capture percentage with *equal sample sizes*. As expected, the 95% confidence interval’s capture percentage varied as sample sizes varied. The 95% confidence interval’s capture percentage was lowest (e.g., 32.9%) when the original study had a large sample size relative to the replication, resulting in a narrow confidence interval. In contrast, the 95% prediction interval’s capture percentage was virtually 95% in all scenarios.

**Table 1:** Capture percentage for means over 50,000 trials.

N	Replication N	95% Confidence Interval Capture Percentage	95% Prediction Interval Capture Percentage
25	25	84.3	95.0
50	50	83.7	94.9
100	100	83.8	95.0
250	250	83.5	94.7
500	500	83.6	95.1
25	50	89.9	95.0
25	100	92.4	95.1
25	250	93.9	95.1
25	500	94.6	95.1
50	25	74.7	94.9
50	100	89.1	94.9
50	250	92.6	94.9
50	500	93.8	94.9
100	25	62.1	94.9
100	50	74.3	94.8
100	250	90.3	95.0
100	500	93.0	95.3
250	25	44.6	95.1
250	50	58.0	95.0
250	100	70.6	94.8
250	500	89.0	95.1
500	25	32.9	94.9
500	50	45.0	95.1
500	100	57.5	95.1
500	250	74.3	95.1

## Prediction Interval for Correlations

We extend the above methods for calculating a prediction interval for means to calculate a prediction interval for a commonly used index of effect size, the correlation coefficient (*r*). Fundamentally, the concept and logic behind the prediction interval’s calculation for a correlation is the same as it is for means. However, because the sampling distributions of non-zero correlations are not normally distributed, a modified procedure must be used to account for distributional asymmetries [34].

To illustrate how we calculate a prediction interval for correlations, consider hypothetical researcher, Richard, who conducts a study (*N*_1_ = 100) and obtains a correlation of *r*_1_ = .35. He wants to conduct a replication of this finding and doubles his sample size to *N*_2_ = 200. To calibrate his own expectations, Richard wants to compute a prediction interval to determine what he can reasonably expect to find in his replication. How might Richard calculate a prediction interval?

To compute a prediction interval for a correlation, we are interested in identifying the expected difference between two correlations (i.e., the original study correlation and the hypothetical replication study correlation) randomly sampled from the same population. We know that, due to sampling error, both Richard’s original study and his, yet-to-be run, replication study, are expected to differ from both the unknown population correlation and from each other in random directions and magnitudes. To estimate how much the two correlations are expected to differ from each other due to sampling error, Richard first needs an estimate of how much two correlations, sampled from the same population, are expected to deviate from each other. Specifically, he needs to calculate the variance of the sampling distribution for the expected difference between two correlations.

When computing the prediction interval for means, we used a derivation of the sampling distribution formula that accommodated different sample sizes in the original and replication study (see Eq 1). In principle, we could use the same approach for correlations that we used for means using Eq 3, below (adapted from [40]). This formula provides the standard error of the difference between two correlations using the *simple asymptotic method* [31]:

$$\begin{array}{cc} SE & =\sqrt{SE_{1}^{2}+SE_{2}^{2}}\\ & =\sqrt{\frac{{\left(1-r_{1}^{2}\right)}^{2}}{N_{1}-1}+\frac{{\left(1-r_{2}^{2}\right)}^{2}}{N_{2}-1}} \end{array}$$ (3)

However, a drawback with this approach is that it assumes the sampling distributions of the original and replicating study are normally distributed. When using correlations, this is not an appropriate assumption as non-zero correlations have asymmetrical sampling distributions. The asymmetry of correlations’ sampling distributions increases as effect sizes increase (i.e., the further the correlations are away from zero). As a result, using the above formula will result in increasingly inaccurate estimates as effect sizes increase.

Fortunately, Zou [34] illustrates how the asymmetry of correlations’ sampling distributions can be taken into account by using what he referred to as a *modified asymptotic method*. He developed this approach to generate a symmetrical confidence interval around the difference between two known correlations, by accommodating the expected asymmetry. A consequence of non-zero correlations having asymmetrical sampling distributions is that when confidence intervals are created for correlations using the *r*-to-*z* approach [41], non-zero correlations may have asymmetrical confidence intervals. Asymmetrical confidence intervals mean that the upper and lower bounds of a confidence interval are not equidistant from the correlation they bound. Zou’s [34] *modified asymptotic method* essentially computes a confidence interval for the difference between two correlations from the two, likely *asymmetrical*, confidence intervals that bound each of the correlations being compared.

We compute the prediction interval for correlations that is able to accommodate asymmetrical sampling distributions by adapting Zou’s [34] approach. Specifically, we create a prediction interval based on a confidence interval, constructed around the known original correlation (*l*_1_, *u*_1_) and a confidence interval constructed around the *unknown*, yet-to-be obtained replication correlation (*l*_2_, *u*_2_). These values are then used in our slightly modified version of Zou’s [34] equations to create the lower-limit (*LL*) and upper-limit (*UL*) of the correlation prediction interval (see Eqs 4 and 5 below). We note that computationally, this prediction interval is the equivalent of calculating a confidence for the difference between two correlations (one real, one imaginary and identical to the first), based on the original and replication sample sizes.

$$\begin{array}{c} \text{Correlation Prediction Interval Lower Limit}\\ =r_{1}-\sqrt{{\left(r_{1}-l_{1}\right)}^{2}+{\left(u_{2}-r_{1}\right)}^{2}} \end{array}$$ (4)

$$\begin{array}{c} \text{Correlation Prediction Interval Upper Limit}\\ =r_{1}+\sqrt{{\left(r_{1}-l_{2}\right)}^{2}+{\left(u_{1}-r_{1}\right)}^{2}} \end{array}$$ (5)

To illustrate how the prediction interval approach works, we revisit Richard’s scenario and calculate a prediction interval using Eqs 4 and 5. We begin by calculating the lower (*l*_1_) and upper (*u*_1_) limits of the 95% confidence interval for Richard’s original correlation (*r*_1_ = .35) using the original sample size (*N*_1_ = 100) via an *r*-to-*z* transformation resulting in a 95% CI [.16, .51]. Without rounding *l*_1_ = .1649195 and *u*_1_ = .5112702. The next step is to create a hypothetical confidence interval for the imaginary replication correlation (*r*_2_), using the standard Fisher *r*-to-*z* process. We assume that both the original study correlation (*r*_1_) and the replication study correlation (*r*_2_) are estimates of the same underlying population correlation. Consequently, the original correlation, *r*_1_, can be used in place of *r*_2_ when calculating the confidence interval for the replication correlation. However, we use the replication sample size to create the confidence interval around this hypothetical correlation (*N*_2_ = 200). To calculate the lower (*l*_2_) and upper (*u*_2_) limits of the 95% confidence interval for Richard’s replication, using the estimated replication correlation (*r*_2_ = .35) and replication sample size (*N*_2_ = 200) via an *r*-to-*z* transformation we find the 95% CI[.22, .47]. Without rounding the values are *l*_2_ = .2220412 and *u*_2_ = .4661072. Lastly, we compute the upper and lower limits of the prediction interval by combining the upper and lower limits of the two previously calculated intervals into the Eqs 4 and 5, above. Doing so results in the following solution:

$$\begin{array}{l} 95\%\text{ Correlation Prediction Interval Lower Limit}\\ =r_{1}-\sqrt{{\left(r_{1}-l_{1}\right)}^{2}+{\left(u_{2}-r_{1}\right)}^{2}}\\ =.35-\sqrt{{\left(.35-.1649195\right)}^{2}+{\left(.4661072-.35\right)}^{2}}\\ =.1315151\\ =.13 \end{array}$$

$$\begin{array}{l} 95\%\text{ Correlation Prediction Interval Upper Limit}\\ =r_{1}+\sqrt{{\left(r_{1}-l_{2}\right)}^{2}+{\left(u_{1}-r_{1}\right)}^{2}}\\ =.35+\sqrt{{\left(.35-.2220412\right)}^{2}+{\left(.5112702-.35\right)}^{2}}\\ =.5558678\\ =.56 \end{array}$$

Thus, for Richard, the prediction interval for his initial study where he found a correlation of .35, (*N*_1_ = 100) if a replication uses a sample size of 200 is 95% PI[.13, .56]. This means that Richard can expect to observe a correlation between .13 and .56, due to sampling error alone. If a replication correlation falls outside the prediction interval, it suggests that factors other than sampling error may be responsible for the deviation.

We note that the approach we present above is not the only method available. Others have recently created an alternate approach to calculate prediction intervals for correlations. Specifically, Patil et al. [31] derived a formula for the correlation prediction interval that addresses the sampling distribution asymmetry issue in an entirely different way. Specifically, Patil et al. [31], used an *r*-to-*z* transformation on the original correlation, then calculated a prediction interval around the z-value using the simple asymptotic method, and then transformed the interval from *z*s back *r*s.

### Capture Performance Test of the Prediction Interval for Correlation

To test the capture percentage performance of the 95% prediction interval and the 95% confidence around the original study’s correlation using Richard’s example above, we followed the same testing procedure we used for means. (1) We obtained a sample (*N*_1_ = 100), from a population with a correlation of ρ = .50 and calculated the “original study” correlation, *r*_1_. (2) Then, we computed a 95% prediction interval and a 95% confidence interval around the original study’s correlation (*r*_1_), using the methods and formulas presented above. (3) We then simulated the replication study by drawing a second random sample (*N*_2_ = 200) and obtained the replication correlation, *r*_2_. (4) We compared the replication correlation (*r*_2_) against the 95% prediction interval and 95% confidence interval to determine if either (or both) captured it. (5) We repeated steps one through four 50,000 times and counted how many prediction intervals and confidence intervals contained the replication correlation. Doing so revealed that the 95% prediction interval captured 94.8% of replication correlations whereas the 95% confidence interval captured only 89.3% of replication correlations.

We also tested the prediction interval and 95% confidence interval’s capture performance using different effect size and sample size configurations. The results of these tests are reported in Table 2. The 95% confidence interval’s capture percentage fluctuated as a function of sample size and, to a small extent, due to the population correlation. The confidence interval captured the fewest replication correlations (e.g., 44.2%) when the original study had a large sample size compared to the replication study. When sample sizes were equal, roughly 83–84% of replications are captured by the 95% confidence interval. In contrast, the 95% prediction interval’s capture percentage was nearly exactly 95% in all scenarios. We note that this was true for sample sizes of 100 or higher. The script to reproduce our correlation capture tests is available at https://github.com/dstanley4/prediction_interval_scripts/.

**Table 2:** Capture percentages for correlations over 50,000 trials.

rho	N	Replication N	95% Confidence Interval Capture Percentage	95% Prediction Interval Capture Percentage
.10	100	100	83.8	94.7
.10	250	250	83.4	94.7
.10	500	500	83.7	94.9
.10	1000	1000	83.4	95.1
.10	100	250	90.5	94.7
.10	100	500	92.7	94.9
.10	100	1000	93.9	95.0
.10	250	100	70.2	94.7
.10	250	500	89.2	94.9
.10	250	1000	91.9	94.7
.10	500	100	57.4	94.8
.10	500	250	74.0	95.0
.10	500	1000	89.0	95.0
.10	1000	100	44.3	94.9
.10	1000	250	61.6	94.9
.10	1000	500	74.6	95.1
.30	100	100	83.4	94.4
.30	250	250	83.2	94.7
.30	500	500	83.6	95.1
.30	1000	1000	83.7	95.1
.30	100	250	90.4	94.8
.30	100	500	92.6	94.7
.30	100	1000	94.0	95.1
.30	250	100	70.3	94.6
.30	250	500	89.0	94.9
.30	250	1000	92.1	94.9
.30	500	100	57.3	94.5
.30	500	250	74.3	94.9
.30	500	1000	89.0	94.9
.30	1000	100	43.9	94.7
.30	1000	250	61.7	94.8
.30	1000	500	74.1	94.9
.50	100	100	83.5	94.7
.50	250	250	83.4	94.9
.50	500	500	83.5	94.9
.50	1000	1000	83.6	95.0
.50	100	250	90.3	95.0
.50	100	500	92.8	95.0
.50	100	1000	93.9	95.0
.50	250	100	70.5	94.5
.50	250	500	89.3	95.1
.50	250	1000	92.1	95.0
.50	500	100	57.3	94.3
.50	500	250	74.2	95.0
.50	500	1000	89.0	94.9
.50	1000	100	44.2	94.3
.50	1000	250	62.0	94.8
.50	1000	500	74.2	95.0

## Prediction Interval for *d*-Values

In this section, we illustrate how the prediction interval can be calculated for another commonly used index of effect size, the standardized mean difference (i.e., *d*-value):

$$d=\frac{M_{1}-M_{2}}{s_{p}}$$

$$s_{p}=\sqrt{\frac{s_{1}^{2}(N_{1}-1)+s_{2}^{2}(N_{2}-1)}{N_{1}+N_{2}-2}}$$

The calculation of a prediction interval for *d*-values follows the same logic and procedure that we used to calculate a prediction interval for correlations because we are, once again, faced with asymmetrical sampling distributions. Specifically, non-zero *d*-values have sampling distributions that follow non-central *t*-distributions and non-central *t*-distributions are asymmetrical. Because of these asymmetrical non-central *t-*distributions, we sought to generalize Zou’s [34] *modified asymptotic* approach to *d*-values. Although Zou [34] only investigated correlations (via *r*-to-*z* transformations) we surmised that his work could be applied to standardized mean differences (via *d*-to-*t* transformations). Specifically, we used the logic behind Zou’s [34] proof to generate a new modified asymptotic formula for *d*-values; however, the resulting equation was identical in nature to the correlation formula, demonstrating that Zou’s [34] formula generalizes to *d*-values. Our application of this *modified asymptotic approach d*-value formula to calculate the prediction interval is presented below in Eqs 6 and 7. We note that computationally, this prediction interval is the equivalent of calculating a confidence interval for the difference between two *d*-values (one real, one imaginary and identical to the first), based on the original and replication sample sizes.

$$\begin{array}{c} d-\text{value Prediction Interval Lower Limit}\\ =d_{1}-\sqrt{{\left(d_{1}-l_{1}\right)}^{2}+{\left(u_{2}-d_{1}\right)}^{2}} \end{array}$$ (6)

$$\begin{array}{c} d-\text{value Prediction Interval Upper Limit}\\ =d_{1}+\sqrt{{\left(d_{1}-l_{2}\right)}^{2}+{\left(u_{1}-d_{1}\right)}^{2}} \end{array}$$ (7)

To illustrate how to compute a prediction interval for *d*-values, we will work through another hypothetical example, this time with hypothetical coffee researcher, Ted. Ted is beginning research on the relation between coffee and attraction to the color red. He expects that drinking coffee will make the color red more appealing. To test his expectation, he conducts an experiment, manipulating coffee intake across two conditions (i.e., a coffee condition and water condition) and recording participants’ preference for the color red. In his study, Ted runs 50 participants in his experimental coffee group and 50 participants in his water control group (i.e., an overall *N*_*original*_ = 100, *N*_*orig*1_ = 50, *N*_*orig*2_ = 50). His results indicated that coffee participants preferred the color red more than those who drank water, *d* = 0.65. Ted is very excited by this finding; however, he wants to follow it up with a replication to confirm his finding before attempting to publish it. To replicate his finding Ted will use the same sample sizes as the original study (i.e., *N*_*replication*_ = 100, *N*_*rep*1_ = 50, *N*_*rep*2_ = 50).

What range of *d*-values can Ted expect to observe in a replication? To answer this question, Ted will need to compute a prediction interval. In doing so, we will be determining how much two random *d*-values sampled from the same population are expected to deviate from one another. Following the modified asymptotic method we used to compute the prediction interval for correlations, Ted first calculates a confidence interval for his original study’s *d*-value. Second, he needs to compute a confidence interval for his yet to be conducted replication study. And lastly, he needs to enter the upper and lower limits of each of these confidence intervals into Eqs 6 and 7, above. As was the case with correlations, this process accommodates the presence of asymmetrical confidence intervals around *d*-values that arise from asymmetrical non-central *t*-distributions [41–43].

Computing a 95% confidence interval for Ted’s original study’s result of *d* = 0.65, with a sample size of 100 (*N*_*orig*1_ = 50, *N*_*orig*2_ = 50) results in 95% CI [0.25, 1.05] (i.e., *l*_1_ = 0.2460344, *u*_1_ = 1.050815). To create a hypothetical confidence interval for Ted’s, yet-to-be observed, replication *d*-value, he assumes that both the original study and the replication study are estimates of the same underlying population *d*-value. Consequently, he can use the original *d*-value (*d*_1_) as his estimate of *d*_*2*_. Doing so results in a 95% CI [0.25, 1.05] (i.e., *l*_2_ = 0.2460344, *u*_2_ = 1.050815) for *d*_2_. Plugging the upper and lower limits for *d*_*1*_ and *d*_*2*_ into our prediction interval formula results in:

$$\begin{array}{l} 95\%\;d-\text{value Prediction Interval Lower Limit}\\ =d_{1}-\sqrt{{\left(d_{1}-l_{1}\right)}^{2}+{\left(u_{2}-d_{1}\right)}^{2}}\\ =0.65-\sqrt{{\left(0.65-0.24603444\right)}^{2}+{\left(1.050815-0.65\right)}^{2}}\\ =0.08093008\\ =0.08 \end{array}$$

$$\begin{array}{l} 95\%\;d-\text{value Prediction Interval Upper Limit}\\ =d_{1}+\sqrt{{\left(d_{1}-l_{2}\right)}^{2}+{\left(u_{1}-d_{1}\right)}^{2}}\\ =0.65+\sqrt{{\left(0.65-0.2460344\right)}^{2}+{\left(1.050815-0.65\right)}^{2}}\\ =1.21907\\ =1.22 \end{array}$$

These values constitute the upper and lower limits of the prediction interval 95% PI[0.08, 1.22]. If Ted’s replication *d*-value differs from the original *d*-value (*d* = .65) due to sampling error alone, he can expect a replication *d*-value between 0.08 and 1.22. Consequently, if a replication *d*-value falls outside the prediction interval factors other than sampling error may be responsible for the deviation.

### Capture Performance Test of the Prediction Interval for *d*-Value

To test the capture percentage performance of the 95% prediction interval and the 95% confidence around the original study’s *d*-value we again conducted a series of simulations. Because a study *d-*value can be biased estimator of the population-level *d-*value, we tested the 95% prediction interval and 95% confidence interval’s capture performance using both *d* and a biased corrected *d* we will term *d*_unbiased_. Bias in this context means that the average of a *d*-value’s sampling distribution does not equal the population *d*-value. Therefore, adjusting the *d*-values to be unbiased (see 44) means that the average of the unbiased sampling distribution of *d*-values equals the population *d*-value. A value for *d*_unbiased_ can be approximated with the equation below [44]. This equation makes the largest adjustment when converting *d* to *d*_unbiased_ when the sample size (i.e., degrees of freedom) is low.

$$d_{unbiased}=d\left(\frac{3}{4(df)-1}\right)$$

The script to reproduce our *d*-value capture tests is available at https://github.com/dstanley4/prediction_interval_scripts/. To test the prediction intervals ability to capture replication *d*-values: (1) We randomly sampled, from two populations that differed by .80, data for two cells (*N*_1_ = 50 and *N*_2_ = 50) and calculated the “original study” standardized mean difference (*d*_1_). (2) We computed a confidence and prediction intervals around the original study’s standardized mean difference (*d*_1_), using the methods and formulas presented above. (3) We then simulated the replication study by drawing a second random sample (*N*_*rep*1_ = 50, *N*_*rep*2_ = 50) and obtained the replication standardized mean difference, *d*_2_. (4) We compared the replication standardized mean difference (*d*_2_) against the 95% prediction interval and 95% confidence interval to determine if either (or both) captured it. (5) We repeated steps one through four 50,000 times and counted how many prediction intervals and confidence intervals contained the replication correlation. This process was followed for both *d* and *d*_unbiased_. For *d*, the 95% prediction interval captured 94.9% of replication correlations whereas the 95% confidence interval captured 83.6% of replication correlations (see script for command). The results were similar for *d*_unbiased_, the 95% prediction interval captured 95.0% of replication correlations whereas the 95% confidence interval captured 83.9% of replication correlations.

We also tested the capture percentage performance of the confidence and prediction interval across a range of cell sizes and population *d*-values (Table 3). The confidence interval performed in the same manner with *d*-values as it did with means and correlations; that is, there was wide variability in capture percentage of the confidence interval across the scenario. The confidence interval captured the fewest replication correlations (e.g., 32.7%) when the original study had a large sample size compared to the replication study. When sample sizes were equal, roughly 83–84% of replications are captured by the 95% confidence interval. When the original study cell sizes were large (e.g., 500) and the replication cell sizes were small (e.g., 25) the prediction interval for the biased *d*-value captured slightly less than 95% of replication results (e.g., 94.4%). However, when *d*_unbiased_ was used, the capture percentage for the 95% prediction interval closely approximated 95% (e.g., 94.8%). Overall, the 95% prediction interval’s capture percentage was approximately 95% in all scenarios.

**Table 3:** Capture percentages for d-values over 50,000 trials.

			Replication		*d*		*d*_unbiased_	
Population *d*	N1	N2	N1	N2	95% Confidence Interval Capture Percentage	95% Prediction Interval Capture Percentage	95% Confidence Interval Capture Percentage	95% Prediction Interval Capture Percentage
0.2	25	25	25	25	83.1	94.9	83.7	95.3
0.2	50	50	50	50	83.1	94.8	83.4	95.0
0.2	100	100	100	100	83.6	95.0	83.8	95.1
0.2	250	250	250	250	83.5	95.0	83.6	95.1
0.2	500	500	500	500	83.3	95.0	83.4	95.1
0.2	25	25	50	50	89.1	95.1	89.5	95.3
0.2	25	25	100	100	92.1	95.1	92.4	95.4
0.2	25	25	250	250	93.9	95.0	94.2	95.3
0.2	25	25	500	500	94.4	95.0	94.8	95.3
0.2	50	50	25	25	73.6	94.7	74.2	95.0
0.2	50	50	100	100	88.8	94.8	89.0	95.0
0.2	50	50	250	250	92.8	95.2	93.0	95.4
0.2	50	50	500	500	93.9	95.0	94.1	95.1
0.2	100	100	25	25	60.9	94.5	61.5	94.8
0.2	100	100	50	50	74.0	94.9	74.3	95.1
0.2	100	100	250	250	90.0	94.9	90.1	95.0
0.2	100	100	500	500	92.7	95.1	92.8	95.1
0.2	250	250	25	25	44.5	94.4	45.1	94.7
0.2	250	250	50	50	57.3	94.7	57.7	94.9
0.2	250	250	100	100	70.3	94.8	70.4	94.9
0.2	250	250	500	500	89.1	95.1	89.1	95.1
0.2	500	500	25	25	33.5	94.4	34.0	94.7
0.2	500	500	50	50	44.5	94.9	44.7	95.1
0.2	500	500	100	100	57.7	95.0	57.9	95.1
0.2	500	500	250	250	74.3	94.9	74.4	95.0
0.5	25	25	25	25	83.0	94.7	83.7	95.1
0.5	50	50	50	50	83.3	94.8	83.6	94.9
0.5	100	100	100	100	83.2	94.9	83.3	95.0
0.5	250	250	250	250	83.4	95.1	83.5	95.1
0.5	500	500	500	500	83.3	95.0	83.4	95.0
0.5	25	25	50	50	89.4	95.1	89.8	95.3
0.5	25	25	100	100	92.2	95.1	92.5	95.3
0.5	25	25	250	250	93.8	95.0	94.2	95.3
0.5	25	25	500	500	94.6	95.1	94.9	95.4
0.5	50	50	25	25	73.8	94.5	74.5	94.8
0.5	50	50	100	100	89.0	94.9	89.2	95.0
0.5	50	50	250	250	92.6	95.0	92.8	95.1
0.5	50	50	500	500	94.0	95.1	94.1	95.2
0.5	100	100	25	25	61.3	94.4	61.8	94.8
0.5	100	100	50	50	74.0	94.8	74.3	95.0
0.5	100	100	250	250	90.5	95.0	90.6	95.1
0.5	100	100	500	500	92.7	95.0	92.7	95.1
0.5	250	250	25	25	44.0	94.6	44.6	95.0
0.5	250	250	50	50	57.3	94.8	57.6	94.9
0.5	250	250	100	100	70.1	94.9	70.2	95.0
0.5	250	250	500	500	89.1	95.0	89.2	95.0
0.5	500	500	25	25	32.8	94.4	33.2	94.8
0.5	500	500	50	50	44.6	94.7	44.8	94.8
0.5	500	500	100	100	57.7	95.0	57.8	95.0
0.5	500	500	250	250	74.3	94.9	74.4	94.9
0.8	25	25	25	25	82.8	94.8	83.4	95.1
0.8	50	50	50	50	83.3	95.0	83.6	95.2
0.8	100	100	100	100	83.3	94.9	83.5	94.9
0.8	250	250	250	250	83.3	95.0	83.4	95.0
0.8	500	500	500	500	83.3	94.9	83.3	94.9
0.8	25	25	50	50	88.7	94.8	89.1	95.1
0.8	25	25	100	100	92.0	95.0	92.3	95.2
0.8	25	25	250	250	93.9	95.1	94.2	95.4
0.8	25	25	500	500	94.4	95.0	94.7	95.2
0.8	50	50	25	25	73.9	94.6	74.5	94.9
0.8	50	50	100	100	89.2	95.0	89.4	95.1
0.8	50	50	250	250	92.5	95.0	92.7	95.1
0.8	50	50	500	500	93.9	95.1	94.1	95.3
0.8	100	100	25	25	61.7	94.6	62.3	95.0
0.8	100	100	50	50	73.5	94.5	73.8	94.7
0.8	100	100	250	250	90.2	95.1	90.3	95.2
0.8	100	100	500	500	92.7	95.0	92.7	95.0
0.8	250	250	25	25	44.0	94.6	44.5	94.9
0.8	250	250	50	50	57.2	94.9	57.4	95.0
0.8	250	250	100	100	70.4	94.8	70.6	94.9
0.8	250	250	500	500	89.0	94.9	89.1	95.0
0.8	500	500	25	25	32.7	94.4	33.1	94.8
0.8	500	500	50	50	44.5	95.0	44.7	95.1
0.8	500	500	100	100	57.8	95.0	58.0	95.1
0.8	500	500	250	250	74.3	95.1	74.4	95.1

## Prediction Interval Calculators

To facilitate easy calculation of the prediction interval, we have created software and a web-based calculator that computes prediction intervals for means, correlations, and *d*-values. The web-based calculators can be found at https://replication.shinyapps.io/mean/ for means, https://replication.shinyapps.io/correlation/ for correlations, and https://replication.shinyapps.io/dvalue/ for *d*-values. We also created a prediction interval package for R, called *predictionInterval*, that users can download for free and use within R. The package is currently available in the R CRAN repository.

Below, are examples of the R commands one would enter to compute the prediction interval for means, correlations, and *d*-values using the *predictionInterval* package. The package would need to be installed, with install.packages("predictionInterval"), and activated, with library(predictionInterval), before using the commands below.

### Mean

> pi.m(M = 98.59,SD = 14.76,n = 50,rep.n = 100)

Original study: M = 98.59, SD = 14.76, N = 50, 95% CI[94.40, 102.78]

Replication study: N = 100

Prediction interval: 95% PI[93.45,103.73].

### Correlation

> pi.r(r = .35,n = 100,rep.n = 200)

Original study: r = 0.35, N = 100, 95% CI[0.16, 0.51]

Replication study: N = 200

Prediction interval: 95% PI[0.13,0.56].

### *d*-Value

> pi.d(d = .65,n1 = 50,n2 = 50,rep.n1 = 100,rep.n2 = 100)

Original study: d = 0.65, N1 = 50, N2 = 50, 95% CI[0.25, 1.05]

Replication study: N1 = 100, N2 = 100

Prediction interval: 95% PI[0.16,1.14].

## Discussion

Replication research is on the rise and a major problem faced by replication researchers is how to decide if the results of a replication are reasonable given the results of a previous study. Artifacts such as sampling error make it so that even the most exquisitely executed and designed studies can be poor reflections of the underlying truth. Consequently, results are expected to vary across replications. Given that variability in results is expected, what range of values is permissible in a replication? The prediction interval is a way to illustrate what range of results can be expected between studies due to sampling error.

Our work extends the literature by deriving prediction interval formulas for means, correlations, and *d*-values that allow for sample sizes differences between the original and replication studies. We addressed the challenge of asymmetrical sampling distributions by deriving formulas based on Zou’s [34] modified asymptotic method. This allows us to calculate prediction intervals for correlations and *d*-values. Additionally, we created an R package and website to facilitate easy calculation of these intervals.

To increase researchers intuitive understanding of the prediction interval (and illustrate it behaves as we claim) we conducted a series of capture simulations. As discussed previously, confidence intervals are frequently misunderstood and often interpreted as prediction intervals (i.e., the confidence-level misconception). Consequently, a key component of our capture tests simulations was contrasting the performance of the prediction interval against that of a traditional confidence interval constructed around a sample mean or effect size estimate. We hope that by presenting these simulations we will increase intuitive understanding of both the prediction interval and the confidence interval. These simulations illustrate the prediction interval’s ability to consistently capture replication results 95% of the time for each statistic (mean, *r*, *d*-value) across a range of sample size configurations, and population effect sizes. In contrast, the 95% confidence interval around the original study’s estimate demonstrated inconsistent performance capturing different percentages of replication results depending on sample size configurations.

In addition to the prediction interval’s ability to accurately capture 95% of replication results that are due to sampling error, the prediction interval has a number of desirable characteristics that are important to highlight. The prediction interval is quantitative, which reduces the need for subjectivity when interpreting replications. It is also easy and intuitive to interpret, which reduces the likelihood that it will be misinterpreted and incorrectly applied. The prediction interval is calculable before a replication study is run, which means its calculation and application will be free of post hoc biases.

We note, however, that a prediction interval should be applied in a thoughtful, rather than rule-based way, when reconciling a replication result with the original study. If the sample sizes of the original and replication study are more or less similar then interpretation of the prediction interval is relatively straight forward as discussed above. However, to the extent that the sample size for the replication study is *substantially* larger than the original study then the prediction interval should be interpreted in a more nuanced manner. Consider a scenario where a small sample size original study (e.g., *r* = .30, *N* = 50) is followed by a very large sample size replication study (e.g., *r* = .04, *N* = 1000). Here the prediction interval for the original study is PI[.02, .54] and the replication correlation, .04, clearly falls within the prediction interval. The fact that the replication correlation, .04, falls within the prediction interval indicates that this much weaker replication correlation may well have occurred due to random sampling alone. Although this is an accurate conclusion, it might not be wise to classify this replication as a “success”. Why not?

Simply put, classifying the *r* = .04 replication as a success implicitly gives too much weight to the original study that observed a moderate effect (*r* = .30) based on a very small sample size (*N* = 50). Calling this replication study a success suggests that the original *r* = .30 effect size estimate was accurate. However, the replication effect size (*r* = .04) seems more likely to provide an accurate estimate of the population correlation given the substantially larger sample size (*N* = 1000; a 20-fold increase). Consequently, in this scenario, the best conclusion may be that there is a weak relation, as indicated by the replication study, and that the original study provided little information.

When interpreting prediction intervals, it is important to keep in mind that the width of the prediction interval is dependent on the sample sizes of both the original and replicating studies. If the sample size of either study is small, the prediction interval will be wide. Consequently, when sample sizes differ substantially across the original and replication studies, interpretation of replication as a success or failure needs to be done in a thoughtful and well-reasoning way that considers the relative sample sizes of the two studies.

### Implications of the Prediction Interval for Programmatic Researchers

We expect that individual researchers conducting programmatic research may benefit from the prediction interval. Consider the scenario in which a researcher begins to investigate a new topic and comes across an interesting finding. A natural next step for the researcher may be to run a follow-up study. If the follow-up produced an effect that deviates substantially from the original study, the researcher might be tempted to abandon research in this new area. In contrast, if this same researcher calculated a prediction interval before conducing the second study, s/he might have a very different reaction to the second finding if s/he could conclude, using the prediction interval, that it was not inconsistent with the first finding. Indeed, in this scenario, the researcher may be more motivated to conduct additional research. In this context, the prediction interval can be used to help researchers operationalize the effect of sample size on the expected variability of results. Specifically, if a prediction interval is computed and is uncomfortably wide, a researcher can increase the sample size of the replication study to narrow the interval. The ease of calculating the prediction interval means that many different sample size configurations can be explored. We hope that this use of prediction intervals will be of substantial value to programmatic researchers moving forward.

### Implications of the Prediction Interval for Registered Replications

The most important implication of the prediction interval is that it can aid in the empirical interpretation of registered replication results. Critically, the prediction interval can be calculated during the planning stage of replication research. This allows researchers, and registered replication reviewers, to recognize the range of results that are possible due to sampling error alone prior to conducting a replication. We suspect *a priori* use of the prediction interval by registered replication reviewers may result in requests for increased replication sample sizes. Ideally, the authors of replication studies would include the prediction interval, but even if they did not, the replication reviewers could calculate it using the tools we have provided. Specifically, replication reviewers would only need to enter the original effect size (*r* or *d*), original sample size, and the proposed replication sample size into our website or R package to obtain the prediction interval.

## Conclusion

We have presented the prediction interval as a way to gauge, before a replication is conducted, how much the replication result may deviate from the original study, due to sampling error alone. The application of the prediction interval to replication research will allow researchers to objectively assess if the result of a replication is statistically consistent or inconsistent with the original finding. Given the history of researchers underestimating the impact of sampling error [45] prediction intervals are a useful tool for calibrating expectations around the precision of interpreting replication research. The prediction interval provides researchers with an objective statistic for interpreting replication results.
